# Involvement of Epigenetic Mechanisms and Non-coding RNAs in Blood-Brain Barrier and Neurovascular Unit Injury and Recovery After Stroke

**DOI:** 10.3389/fnins.2019.00864

**Published:** 2019-08-20

**Authors:** Svetlana M. Stamatovic, Chelsea M. Phillips, Gabriela Martinez-Revollar, Richard F. Keep, Anuska V. Andjelkovic

**Affiliations:** ^1^Department of Pathology, University of Michigan Medical School, Ann Arbor, MI, United States; ^2^Neuroscience Graduate Program, University of Michigan Medical School, Ann Arbor, MI, United States; ^3^Department of Neurosurgery, University of Michigan Medical School, Ann Arbor, MI, United States; ^4^Department of Molecular Integrative Physiology, University of Michigan Medical School, Ann Arbor, MI, United States

**Keywords:** blood brain barrier, neurovascular unit, stroke injury, inflammation, DNA methylation, histone deacetylases, microRNA, non-coding RNA

## Abstract

Cessation of blood flow leads to a complex cascade of pathophysiological events at the blood-vascular-parenchymal interface which evolves over time and space, and results in damage to neural cells and edema formation. Cerebral ischemic injury evokes a profound and deleterious upregulation in inflammation and triggers multiple cell death pathways, but it also induces a series of the events associated with regenerative responses, including vascular remodeling, angiogenesis, and neurogenesis. Emerging evidence suggests that epigenetic reprograming could play a pivotal role in ongoing post-stroke neurovascular unit (NVU) changes and recovery. This review summarizes current knowledge about post-stroke recovery processes at the NVU, as well as epigenetic mechanisms and modifiers (e.g., DNA methylation, histone modifying enzymes and microRNAs) associated with stroke injury, and NVU repair. It also discusses novel drug targets and therapeutic strategies for enhancing post-stroke recovery.

## Introduction

Stroke is defined as an abrupt onset of focal or global neurological symptoms caused by a blockage of cerebral vessels (ischemic stroke), rupture of blood vessels (hemorrhage), or transient occlusion of small blood vessels (transient ischemic attack). It is particularly prevalent in the aging population, with people over 65 years old accounting for ∼75% of all registered cases (Hollander et al., 2003). Ischemic stroke is further subdivided based on the caliber of occluded vessels into macro- and microvascular (i.e., lacuna stroke), and based on the origin of clot-causing blockage into (a) thrombotic stroke where clot form inside brain blood vessels, and (b) thromboembolic/embolic stroke where clots form elsewhere in the body and travel toward and lodge in brain blood vessels (Reed et al., 2014; Topcuoglu et al., 2018; Tsai et al., 2018).

Pathophysiologically, cessation of blood flow leads to a complex cascade of events at the blood-vascular-parenchymal interface which evolves over time and space, and results in damage to neural cells and edema formation (Dirnagl et al., 1999; Allen et al., 2012). The central events in the hyperacute phase (within minutes and up to 6 h) include compromised mitochondrial function, anaerobic glycolysis, decreased pH (acid condition), impaired ATP production and reduced ion pump activity. As a consequence, cells swell and die, predominantly due to accumulation of lactic acid, ions (Ca^2+^ and Na^+^) and increased water influx (Dirnagl et al., 1999; Ginsberg, 2008; Nagy and Nardai, 2017).

There follows a cascade of events in the acute and subacute phase (hours to 7 days), including blood-brain barrier (BBB), and neurovascular unit (NVU) damage characterized by mitochondrial failure, robust production of reactive oxygen species (ROS) (superoxide O_2_, hydrogen peroxide, and peroxynitrite), excitotoxicity (release of glutamate from dying neurons), activation of matrix metalloproteinases (MMP2, astrocytes; MMP3, MMP9 endothelial cells and neutrophils), BBB damage triggering inflammation and blood cell infiltration (neutrophils, monocytes) that can lead to further cell death, and cellular and vasogenic edema (Enzmann et al., 2013; Posada-Duque et al., 2014; Sifat et al., 2017). The secondary damage mostly takes place in the penumbra surrounding the core infarct and its progression can extend into the chronic phase after stroke.

Paralleling these injury processes, there is activation of endogenous protective and repair mechanisms that include vascular remodeling, angiogenesis, and neurogenesis (Chopp et al., 2007; Venna et al., 2014). The degree of these ongoing repair processes on one side and persistent inflammation/damaging processes on the other determines stroke recovery and the potential risk of another stroke.

There is mounting evidence on the importance of epigenetic factors in stroke. The purpose of this review is to examine how such factors impact the cerebrovasculature in stroke injury and recovery, focusing on effects at the BBB, and NVU. There is debate over whether non-coding RNAs should be included as an epigenetic mechanism and this review will cover their effects as well as other epigenetic mechanisms.

## The Blood-Brain Barrier and Neurovascular Unit in Stroke

The blood-brain barrier is a highly complex and dynamic barrier, formed by an interdependent network of brain capillary endothelial cells, endowed with barrier properties. The BBB strictly regulates paracellular permeability due to the presence of tight junctions (TJs) between endothelial cells. Those TJs are built by intricate interactions between transmembrane proteins (claudins -5, -3, -1, -12, occludin, and JAM-A), important for paracellular space occlusion, scaffolding proteins (ZO-1, -2), and the actin cytoskeleton vital for physical support and TJ function (Daneman et al., 2010; Stamatovic et al., 2016). The transcellular interactions of claudin-5 play the major role in occluding the paracellular space (Nitta et al., 2003; Ohtsuki et al., 2007). Any loosening of its adhesive interactions directly affects BBB integrity and increases paracellular permeability. The BBB role of other claudins with lower expression is still uncertain and under investigation (Tietz and Engelhardt, 2015; Sladojevic et al., 2019). BBB function is also dependent on the perivascular microenvironment, which contains cells (e.g., pericytes, astrocytes, and perivascular macrophages), neuronal endings and tissue unique extracellular matrix (Mae et al., 2011; Muoio et al., 2014). Because of this functional integration, nearly two decades ago, the concept of a BBB was broadened to a new structure, the NVU.

The neurovascular unit is composed of BBB-endowed endothelial cells and a perivascular milieu composed from cells including pericytes, smooth muscle cells, astrocytes, perivascular macrophages/microglia, neurons/neuronal endings, and extracellular matrix. The NVU mediates neurovascular coupling, modulating vessel tone (Mae et al., 2011; Muoio et al., 2014). These intimately and reciprocally linked cells and matrix generate a complex structure that regulates exchange between blood and brain, oxygen and nutrient delivery, and regional cerebral blood flow. It is essential for maintaining circulatory and brain homeostasis.

In stroke, blood-brain barrier, and neurovascular unit dysfunction actively contributes to injury pathogenesis, being a “solid substrate” for ongoing injuring processes (oxidative stress, inflammation, and cytotoxicity), contributing to ischemic core (infarct) formation in the acute phase of stroke, and facilitating the progression of injury in the subacute and chronic phases. For example, in the early (acute) phase of stroke, NVU dysfunction is characterized by disruption of BBB integrity/BBB breakdown (disassembly of TJ complex, decreases in the TJ proteins claudin-5, occludin, and ZO-1) that leads to vasogenic brain edema, a life-threatening acute stroke complication (Bauer et al., 2010; Zehendner et al., 2011; Sladojevic et al., 2014). The cell components of the NVU undergo a series of transformations during injury. Brain endothelial cells, for example, are affected very early by cytotoxic effects with dysfunction of ion channels and transporters (e.g., Na^+^-K^+^-Cl^–^ cotransporter, and Na^+^/H^+^ exchanger), release of extracellular vesicles and conversion of brain endothelial cells toward a proinflammatory and prothrombotic phenotype due to upregulation of protease-activated receptor 1 (PAR-1), tissue factor, and matrix metalloproteinases (MMPs) (Zhu et al., 2008; Bauer et al., 2010; Yamashita and Abe, 2011; Chen et al., 2015). The proinflammatory phenotype of brain endothelial cells involves an upregulation of endothelial adhesive molecules (ICAM-1, VCAM-1, P-, and E- selectins) that guide leukocyte infiltration in the acute inflammatory phase response and T and B cells infiltration in the late phase (Kleinschnitz et al., 2013; Zhou et al., 2013; Sladojevic et al., 2014). Overall, inflammation is thought to worsen acute ischemic injury, contributing to chronic focal inflammation and restricting functional recovery. However, inflammation is also involved in tissue repair.

In the hyperacute phase after stroke, pericytes may be involved in vasoconstriction, causing capillary occlusion (no-reflow phenomenon), while later, by switching to pro-inflammatory phenotype, they may enhance BBB permeability, and brain edema formation (Hall et al., 2014; Underly et al., 2017). Ischemia triggers a series of damaging reactions in astrocytes including mitochondrial dysfunction, energy depletion, ion disequilibrium, increased glutamate and Ca^2+^, aquaporin 4 (AQP4) channel activation, increased water permeability, and cell swelling (Friedman et al., 2009; Ito et al., 2009; Hertz et al., 2014; Mogoanta et al., 2014). It results in the release of oxidative stress products and inflammatory cytokines/chemokines (IL1, IL6, IL15, CCL2, CXCL1, CXCL10, CXCL12, and IP-10), proinflammatory associated small molecules [S100 Ca^2+^-binding protein B (S100B) and nitric oxide (NO)] that enhance the inflammatory post-stroke response (Yamashita et al., 2000; Hill et al., 2004; Mori et al., 2008; Shin et al., 2014; Liu H. et al., 2015; Chen et al., 2018).

**FIGURE 1:**
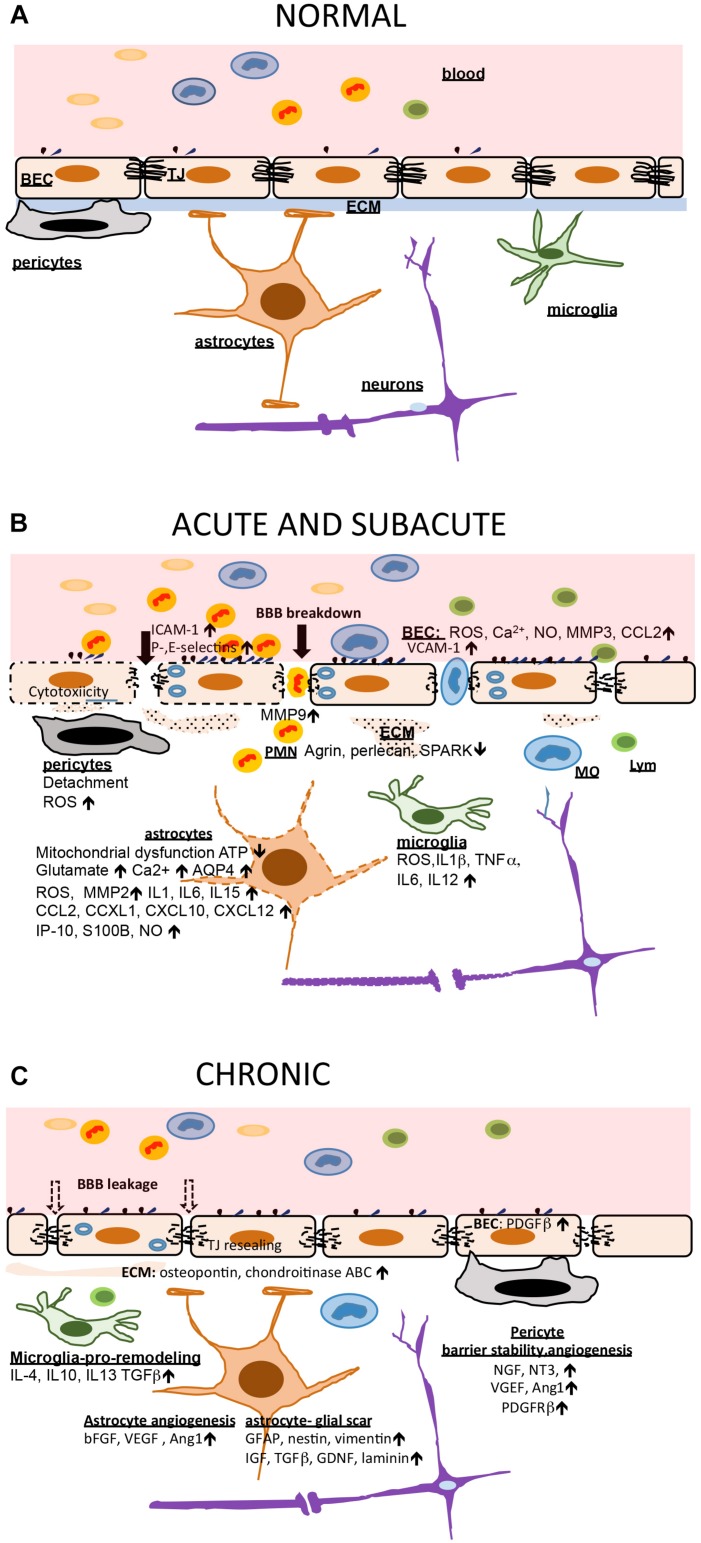
Blood brain barrier (BBB) and neurovascular unit (NVU) in ischemic injury and post-stroke recovery. **(A)** In healthy conditions, the BBB is intact and other NVU components, including the extracellular matrix (ECM), support and preserve brain homeostasis. **(B)** Cessation of blood flow triggers a chain reaction at the BBB and NVU. The early events are mostly characterized by cytotoxicity, mitochondrial dysfunction and accumulation of ROS which further cause BBB breakdown (tight junction, TJ, disruption), neuronal injury guided by astrocytes, and triggering an acute inflammatory response. Brain endothelial cells (BEC) increase adhesion receptor expression allowing leukocyte (predominantly polymorphonuclear neutrophils; PMNs) entry which adds to BBB injury. Microglia and astrocytes produced large amount of proinflammatory cytokines and chemokines amplifying inflammation. Early pericyte detachment support BBB instability and breakdown. This support vasogenic brain edema formation. **(C)** The subacute and chronic phase of stroke is characterized by increased second wave of inflammation with monocyte (MO) and lymphocyte (Lym) entry but also ongoing repair processes (BBB recovery and angiogenesis). Microglia become source of anti-inflammatory cytokines and have a role in phagocytosing dead cells. Astrocytes are a source of growth factors supporting angiogenesis, and also source of extracellular matrix building a gliotic scar. Pericytes establish interactions with BEC, supporting barrier stabilization and new vessel formation. The BBB, with new TJ protein synthesis, undergo partial sealing.

Perivascular macrophages and microglia play an important role in the stroke-induced inflammatory response via production of proinflammatory cytokines (IL1|*upbeta* TNFα IL6, IL12) and ROS (Drake et al., 2011; Liu H. et al., 2015; Wu et al., 2016; He et al., 2019). They trigger the first line of inflammation at the NVU in the acute phase of stroke. Notable changes also occur in the extracellular matrix. At early time points (within hours), there is MMP-related basement membrane degradation with reductions in agrin, SPARC, perlecan, laminin, and fibronectin (Sole et al., 2004; Castellanos et al., 2007; Lee et al., 2011; Ji and Tsirka, 2012; Lloyd-Burton et al., 2013). This ultimately leads to increased BBB disruption, accumulation of new extracellular matrix proteins (i.e., chondroitin sulfate proteoglycan neurocan and osteopontin) and leakage of plasma proteins, such as fibrinogen, into the CNS. This mediates inflammation, edema, and potentially hemorrhagic transformation (Figure 1).

## The Blood-Brain Barrier and Neurovascular Unit in Stroke Recovery

In post-stroke conditions, the NVU has the ability and capacity for remodeling, and this is becoming a very important therapeutic target for enhancing stroke recovery. Remodeling involves complex and tightly tuned interactions between neurons, glial and brain endothelial cells, recruitment of endothelial and neural progenitor cells, and inflammatory blood cells (monocytes, T and B lymphocytes), governing new blood vessel formation, glial cell remodeling of extracellular matrix, for augmented improvement of the NVU, and neurological recovery.

Blood-brain barrier recovery involves *de novo* synthesis of junctional proteins and reestablishing barrier integrity to reduce further brain damage. It is important to highlight that BBB recovery is limited and complete pre-stroke impermeability is difficult to achieve. Ongoing angiogenic processes, as well as defects in the structural repair (e.g., imbalance in the synthesis of claudins essential for TJ function) play a role in the prolonged BBB leakiness days after stroke (Yang Y. et al., 2015; Xu H. et al., 2017; Sladojevic et al., 2019). Whether there are benefits of BBB post-stroke leakage is still a controversial issue. From the perspective of stroke treatment, it may facilitate brain drug delivery. However, it may allow uncontrolled entry of blood components into brain fueling inflammation.

In neurovascular unit remodeling after stroke, cellular elements have important roles in recovery. Pericytes are a source of neurotrophins and have a role in stabilizing the BBB and protecting brain parenchyma from leukocyte infiltration (Shimizu et al., 2012; Yang et al., 2017). They also promote angiogenesis and neurogenesis. Astrocytes undergo structural and functional transformation (reactive gliosis), manifested as increased expression of the intermediate filament protein glial fibrillary acidic protein (GFAP), cell proliferation, and synthesis of extracellular matrix to form the glial scar and demarcate the infarct necrotic core. Higher production of GFAP, nestin and vimentin in NVU negatively affect cell-cell communication at NVU during the subacute and chronic phase of stroke, while production of insulin-like growth factor, transforming growth factor β (TGFβ), and other growth factors as well as laminin by astrocytes enhance NVU recovery processes (Cekanaviciute et al., 2014; Yao et al., 2014; Okoreeh et al., 2017). During post-stroke NVU recovery, microglia transform to a M2 pro-remodeling phenotype, releasing anti-inflammatory cytokines (e.g., IL10) and growth factors. They switch astrocytes to anti-inflammatory phenotype, supporting clean-up phagocytosis, diminishing leukocyte-endothelial cells interaction, decreasing expression and activation of cytokine receptors, promoting NO-induced vasodilatation, and reducing ROS production and oxidative stress by inhibiting NADPH oxidase (Weston et al., 2013; Brifault et al., 2015; Yang Y. et al., 2015).

The extracellular matrix also plays an important role in NVU remodeling and stroke recovery. Interaction between the matrix and its receptors on NVU cells regulate cell survival, migration and focal bioavailability of growth factors essential for repair processes, including angiogenesis and neurogenesis. For example, while osteopontin is normally produced in development, its activity post-stroke, via αβ3 receptors on astrocytes, promotes astrocyte migration and glial scar formation (Ellison et al., 1999; Suzuki et al., 2010). On the other hand, fibrinogen shifts its mechanism of action from the αβ3 receptor to regulating transforming growth factor -β on astrocytes promoting reactive astrocytosis (Schachtrup et al., 2010; Beck and Schachtrup, 2012). Synthesis and upregulation of new extracellular matrix components like chondroitinase ABC may rescue the “injury type” of extracellular matrix by degrading proteoglycans (neurocan) that affect neuronal growth to further enhance the recovery processes (Hill et al., 2012; Chen et al., 2014; Figure 1).

Overall, targeting the NVU in stroke may have long lasting effects by preventing tissue damage in the early hours after stroke and enhancing ongoing recovery processes chronically after stroke. It is, however, very important to highlight that the functional response of NVU cells to stroke as well as the degree of recovery post-stroke will depend on the functional status of the NVU before the stroke which is affected by many co-morbidities (e.g., age, diabetes and hypertension).

## Epigenetic Regulation of BBB and NVU Recovery After Stroke

An overview of decades of stroke research and a variety of identified targets, has unfortunately highlighted that current therapeutic approaches to limit ischemic brain injury have almost all failed, the exception being reperfusion. In recent years, approaches have started to shift toward preventing injury progression and enhancing repair and regeneration after stroke. Targets include inflammation and injury progression, neuronal repair to prevent ischemic cell-death and cerebrovascular remodeling that may prevent further injury progression. The NVU has a focal position in these new approaches to treat stroke.

How to approach treatment of stroke recovery when many conventional approaches (e.g., anti-oxidative, anti-inflammatory) have failed? It has to be taken into consideration that the recovery process is complex and tightly regulated. Many genes are involved in the temporal and spatial regulation of restoration of function with a high degree of individual variation that may depend upon multiple factors. An emerging field, epigenetics, holds promise for a better understanding of what regulates recovery after stroke and the potential success of modulating that process.

Epigenetics is defined as the interaction between environmental factors and the genome that may be heritable and modified gene expression or phenotype without changes in DNA sequences (Hendrich and Willard, 1995). Epigenetic mechanisms (e.g., DNA methylation, post-translational modification of histones) provide an additional level of tight control at the transcriptional level that differentially modifies gene expression and protein activity. In addition, cells possess a powerful machinery in non-coding RNAs, particularly microRNAs (miRs), that regulates gene expression at the post-transcriptional level. Coordinate and cooperative activity of epigenetics factors and miRs form circuits that regulate gene expression profiles and support cell function. Considering the far-reaching effects of epigenetics and miRs on gene regulation, these factors may have a key role in regulating plasticity during repair after stroke and be a key target for enhancing NVU recovery. In this review, we highlight some of the mechanisms that take place at the NVU and are involved in epigenetic and miRs remodeling of the NVU in stroke.

## DNA Methylation

DNA methylation is an epigenetic mechanism characterized by covalent addition of methyl (CH_3_) group to DNA that modifies gene function and expression. The best characterized methylation process occurs on the 5-carbon of the cytosine ring resulting in 5-methylcytosine (5-mC). 5-mC is found exclusively as paired symmetrical methylation of a CpG site where cytosine nucleotide is located next to a guanidine nucleotide (Zeng and Chen, 2019). In genomic DNA, most CpG sites are methylated while CpG islands (clusters) located in proximity to promoter regions endure unmethylated allowing the regulation of gene expression, transcriptional repression (Bird, 1986). DNA methylation occurs via a family of enzymes, the DNA methyltransferases (DNMTs). Five types of DNA methyltransferases have been described: DNMT1- maintains existing methylation patterns following DNA replication, DNMT2 -which can act as a nuclear as well tRNA methytransferase, DNMT3a and DNMT3b responsible for establishing *de novo* methylation patterns and DNMT3L which does not mediate methylation but interacts with histone deacetylase 1 (HDAC1) (Hsieh, 1999; Aapola et al., 2002; Goll and Bestor, 2005; Goll et al., 2006; Ren et al., 2018). It is also important to highlight that DNMT may regulate the expression of certain miRs by direct methylation of miR promoter regions, adding additional layer of DNMT activity at the post-transcriptional level via miRs (Dakhlallah et al., 2013; Nicholson et al., 2017).

Early research investigating epigenetic changes following middle cerebral artery occlusion (MCAO) in mice demonstrated upregulated global DNA methylation within the brain, and genetic or pharmacologic DNMT inhibition decreased stroke severity (Endres et al., 2000). Within the NVU, altered DNA methylation is associated with both BBB injury and recovery, suggesting a biphasic pattern of post-stroke DNA methylation. Mouse MCAO leads to hypermethylation of the tissue inhibitor of metalloproteinase 2 (TIMP2) promoter. The resulting decrease in TIMP2 expression is associated with increased matrix metalloproteinase 9 (MMP9) activity and BBB hyperpermeability, with DNMT inhibition rescuing BBB permeability (Mondal et al., 2019). However, increased DNMT activity post-stroke is also implicated in angiogenesis. Oxygen-glucose deprivation (OGD) in cerebral endothelial cells promotes hypermethylation of the thrombospondin-1 promoter. As thrombospondin-1 inhibits angiogenesis, such promoter hypermethylation suggests DNMT activity promotes post-stroke angiogenesis (Hu et al., 2006). While research investigating post-stroke DNMT activity within the NVU is limited, current studies suggest a dual role of DNMTs within the NVU. An intriguing recent clinical study by Baccarelli et al. (2010) found that blood DNA hypomethylation was associated with a high risk stroke occurrence and total mortality, suggesting that DNMT activity could be a potential biomarker in post-stroke recovery.

To fully delineate the effects of post-stroke DNMT activity, future research must investigate: (1) specific genes with altered methylation patterns, and (2) the pattern of DNA methylation over time. As post-stroke DNMT activity is implicated in both BBB injury and recovery, investigating post-stroke methylation temporal patterns will begin to elucidate the complex roles of DNMTs in ischemic stroke.

## Histone Modifications

DNA wraps around histones forming nucleosomes and creating organized chromatin. Histones are octameric proteins containing modifiable N-terminal residues and post-translational histone modifications alter chromatin accessibility, leading to either transcriptional activation or repression. One well-characterized histone modification is the addition or removal of an acetyl group to lysine residues. Generally, histone acetyltransferases (HATs) add acetyl groups to lysine residues allowing transcription, as the acetyl group disrupts the interaction between the histone residue and DNA. Histone deacetylases (HDACs) remove acetyl groups, leading to transcriptional repression. Ultimately, a balance between HAT and HDAC activity regulates histone acetylation (Narlikar et al., 2002; Li et al., 2007; Kassis et al., 2017). Another form of histone modification is methylation. Methyl group are added on histone N terminal tails at either lysine or arginine residues (Klose and Zhang, 2007). The combination of methylation/demethylation pattern and interplay between lysine or arginine methylases and demethytransferases governs the transcriptional activation/repression of certain genes (Klose and Zhang, 2007; Migliori et al., 2010).

The HDAC superfamily consists of 18 different subtypes in four different classes. Expression of different HDACs within the brain varies by cell type, and mouse MCAO alters HDAC expression in a cell-specific manner (Baltan et al., 2011). Within the NVU, ischemic stroke upregulates astrocytic expression of HDAC2 and HDAC8 (Baltan et al., 2011; Demyanenko et al., 2018). Interestingly, ischemic stroke prominently upregulates HDAC2 in astrocytic processes and end-feet, suggesting HDAC2 affects the NVU post-stroke (Baltan et al., 2011). Current research has demonstrated that increased HDAC expression leads to BBB injury, as HDAC inhibition rescues BBB permeability observed in rodent models of ischemic stroke, including those with increased stroke severity (Park and Sohrabji, 2016; Shi et al., 2016). OGD upregulates endothelial HDAC9 expression and is associated with decreased expression of TJ proteins, such as ZO-1, claudin-5, and occludin. Genetically targeting HDAC9 pre-OGD rescues altered TJ protein expression in endothelial cells (Shi et al., 2016). Other studies indicate post-stroke HDAC expression induces inflammation and oxidative injury. Although contextualized within post-stroke neuroprotection, these studies potentially indicate a mechanism through which altered HDAC expression contributes to barrier injury as well (Wang et al., 2012; Patnala et al., 2017). HDAC inhibitors show a variety of effects after stroke. Valproic acid and sodium butyrate (class I, IIa and III inhibitor) repressed the nuclear translocation of NFκB subunit p65, mitigate MMP9 activity and restore the BBB integrity after stroke by regulating the expression of tight junction proteins, claudin-5, and ZO-1 (Wang et al., 2011; Park and Sohrabji, 2016). Non-specific HDAC inhibition also switches the pro-inflammatory microglial response to anti-inflammatory (Patnala et al., 2017). Likewise, pre-OGD HDAC inhibition promotes anti-oxidative enzyme expression in macrophages in a histone-independent manner (Wang et al., 2012).

While most studies focus on enzyme-mediated changes in histone acetylation, another mechanism through which histone acetylation may be altered in ischemic stroke is through histone eviction. Exposing endothelial cells to hypoxic conditions results in histone eviction from specific promoter regions, such as the eNOS promoter, and replacement with hypoacetylated histones (Fish et al., 2010). Overall, studies investigating the role of histone acetylation in post-stroke recovery indicate that increased HDAC expression and histone hypoacetylation promotes BBB injury.

The specific pattern of histone methylation, and activity of histone lysine methylases and demethylases are important for stroke occurrence and regulation of stroke injury and recovery (Chisholm et al., 2015; Miao et al., 2015; Chakravarty et al., 2017). For example, the pattern of histone 3 methylation of lysine 4 or 9 residues in astrocytes, regulates different gene expression and may regulate the severity of stroke in rodents (Chisholm et al., 2015). The activity of histone lysine methylases (G9a lysine methylases) and demethylases (KDM4B lysine demethylases) was linked to inflammation by regulating TNFa mediated ICAM-1 and VCAM-1 expression in cerebral blood vessels and neutrophil infiltration (Choi et al., 2017). In addition, TNFa serum levels were closely correlated with H3K9Ac and H3K4me3 activity, suggesting an effect on stroke outcomes (Gomez-Uriz et al., 2014). Histone arginine methylation also plays a role in the inflammatory response after stroke. Hypomethylation of histone arginine residues and low asymmetric dimethylarginine (ADMA) levels are associated with increased inflammation and stroke risk in a hyperhomocysteinemia rodent model (Esse et al., 2013). Furthermore, ADMA and symmetric dimethylarginine (SDMA) expression levels were reported to be linked with inflammatory mediator expression (CCL2, IL6, S100B, MMP9, and tissue inhibitor of matrix metalloproteinase-1) and the expression of these mediators and inflammation closely correlated with histone arginine methylation (Chen et al., 2012). ADMA levels are potential biomarkers for stroke injury.

Multiple HDAC inhibitors have already undergone clinical testing for a variety of diseases, with vorinostat approved for treating T cell lymphoma and panobinostat for multiple myeloma (McClure et al., 2018). A non-specific HDAC inhibitor, valproic acid, has been used for many years in the treatment of epilepsy, although there are questions over whether those effects are mediated by HDAC inhibition. The knowledge from those clinical trials may help facilitate translation of HDAC inhibitors to the clinic, as may the generation of new inhibitors of specific HDAC subtypes that might lessen drug side effects (Cao et al., 2018).

Intriguing candidates for regulating BBB and NVU recovery after stroke are the sirtuins. Sirtuins are nicotinamide adenine dinucleotide (NAD^+^) dependent class III histone deacetylases, first discovered in *Saccharomyces cerevisiae* as silent information regulator 2 (Sir2). There are seven mammalian sirtuins homologs (Sirt1-7) localized in nucleus (Sirt1, Sirt6, and Sirt7), mitochondria (Sirt3, Sirt4, and Sirt5), and cytoplasm (Sirt2). Generally, they are implicated in regulating aging, metabolism and cell cycle (Inoue et al., 2007). Besides targeting histones, sirtuins (i.e., Sirt1) can interact with specific DNA binding transcription factors and coregulators (i.e., FOXO, hypoxia inducible factor 1a, PGC1a) and deacetylation of these non-histone targets are responsible for transcriptional regulation and both negative and positive regulation of target gene expression (Andreou et al., 2012; Shukla et al., 2016; Chen et al., 2019).

Epigenetic roles of sirtuins, predominantly Sirt1 and 3, are described in stroke injury via acetylation/deacetylation of targets. Limited data highlight the role of Sirt1 in neuroprotection in the settings of stroke by deacetylation and subsequent inhibition of p53- and NFkB-induced inflammatory and apoptotic pathways, regulation of microglia activity via CX3CR1, brain endothelial cell protection by inhibiting nitric oxide synthesis, and balancing Sirt3 (mitochondrial Sirt) activation after stroke injury (Yang F. et al., 2015; Sellner et al., 2016; Chen T. et al., 2018). Resveratrol, a potent activator of Sirt1, has neuroprotective effects after stroke (enhancing neurite outgrowth and synaptogenesis) and vascular protective effects, reducing the chance of recurrent stroke (Tang et al., 2017). In addition, Sirt1 regulates BBB integrity in aging and CNS infection (Castro et al., 2016; Stamatovic et al., 2019). Although the role of sirtuins in NVU and BBB recovery after stroke is still largely unexplored, emerging data on sirtuin effects on the BBB, endothelial cells, and stroke, put these molecules, and particularly Sirt1, as important targets for epigenetic modification of NVU and BBB in stroke recovery (Table 1).

**TABLE 1:** NVU HDAC cell expression profile and effect in post-stroke recovery.

	**NVU cells**	**Target**	**Effect**	**References**
	**HDAC class I**			
HDAC1	Neurons	?	Neurotoxic	Formisano et al., 2015; Demyanenko et al., 2018
HDAC2	Neurons Astrocytes	?PSD-95, synapsin	Regulate neuroplasticity	Formisano et al., 2015; Tang et al., 2017
HDAC3	Neurons	?	Neurotoxic	Chen et al., 2012
HDAC8	Microglia	?MMP9, Cox2/iNOS	Microglia	Demyanenko et al., 2018
	**HDAC class II (a and b)**			
HDAC4 (a)	Neurons BEC	HMBG1 HIF- a, VEGF CREB Nox4, MMP9	Neuronal death Angiogenesis Neurogenesis Tight junctions	He et al., 2013; Kassis et al., 2015; Zhang Q.-Y. et al., 2017
HDAC5 (a)	Neurons	Neurotoxic (HMBG1)		He et al., 2013
HDAC7 (a)	Undefined			
HDAC9 (a)	BEC	Tight junction proteins Cox2/iNOS, NFkB	BBB integrity Inflammation	Shi et al., 2016; Lu et al., 2018
HDAC6 (b)	Neurons	Nrf2	Oxidative stress	Gaisina et al., 2018
HDAC10 (b)	Undefined			
	**HDAC class III**			
SIRT1	BEC, astrocytes		BBB integrity angiogenesis	Zheng et al., 2018; Sun et al., 2019; Xu et al., 2019
SIRT2	Neurons	FOXO3a	Apoptosis	She et al., 2018
SIRT3	BEC, astrocytes, microglial	p53, NFkB	Oxidative stress	Chen T. et al., 2018; Yang et al., 2018
SIRT4	Neurons	FOXO	Neurotoxic	Sangaletti et al., 2017
SIRT5	BEC		Diminish BBB integrity	Diaz-Cañestro et al., 2018
SIRT6	Neurons	Nrf2		Zhang W. et al., 2017
SIRT7	Undefined	?	Post-stroke biomarker	Wong et al., 2015
	**HDAC class IV**			
HDAC11	Undefined	?	Neurotoxic ?	Chen et al., 2012

*BEC, brain endothelial cells; NVU, neurovascular unit; HDAC, histone deacetylase; Sirt, sirtuin; PSD-95, postsynaptic density protein 95; HMBG1, high-mobility group protein 1; FOXO, forkhead box protein; HIF-a, hypoxia inducible factor-a; CREB, cAMP response element binding protein.*

To determine the mechanisms leading to BBB injury in ischemic stroke, future research should determine which genes have altered expression due to aberrant HDAC activity. Because both HDAC and HAT activity determine histone acetylation patterns, post-stroke HAT activity within the NVU also needs to be determined.

## Non-Coding RNAs (ncRNAs)

Non-coding RNAs represent a group of untranslated regulatory RNA molecules able to modify one or more genes/protein function affecting multiple cellular pathways in biological processes. Non-coding RNAs include ribosomal RNA (rRNA), transfer RNA (tRNA), small (<200 nucleotides), and long (>200 nucleotides) RNAs. Small ncRNA include large group of miRs, piwi-interacting RNAs (piRNA), small nuclear RNAs (snRNA), small nucleolar RNAs (snoRNA), and promoter associated small RNAs. Long ncRNAs are divided into long intergenic ncRNAs, long iontronic ncRNAs, telomeric ncRNAs, pseudogene transcripts, enhancer RNAs and promoter associated long RNAs. Small and long ncRNAs differ in origin, processing and mechanisms of action (Wei et al., 2017).

## MicroRNAs

MicroRNAs are a class of short (∼20–25 nucleotides) non-coding RNAs capable of regulating gene/protein expression by inhibiting translation. Located in cytoplasm, miRs build RNA-induced silencing complexes (RISCs) by binding with Argonaute (Ago) proteins. Direct binding of miR-RISCs to complementary and unique 3′ UTR (untranslated) sequence motifs on target mRNAs, leads to sequestration or degradation of the target mRNA and translational silencing. miRs have multivalent properties, targeting multiple genes, and regulating protein expression in several key cellular processes (cell differentiation, cell cycle progression and apoptosis) (Ambros, 2001; Vishnoi and Rani, 2017). miR expression and function is closely associated with and dependent on interaction with epigenetic factors (DMNT and HDAC) (Dakhlallah et al., 2013; Lee et al., 2017; Nicholson et al., 2017). Together they form complex circuits for transcriptional and post-transcriptional regulation of genes. To date, ∼2000 miR family members have been identified in human, and it is assumed that miRs could regulate ∼60% of all mammalian mRNAs. The family classification is based on a common miR ancestor (Vishnoi and Rani, 2017). Family members share similar physiological function but not always primary sequence or secondary structure. miRs are linked to many disease conditions and represent both potential therapeutic targets but also biomarkers.

MicroRNA are highly differentially expressed in brain tissue and they play key roles in development, physiology, and disease. Cerebral ischemia triggers selective and temporally regulated miR expression that regulate stress responses (e.g., excitotoxicity, oxidative stress, and apoptosis) as well as a spectrum of other processes (e.g., transcription, inflammation, and angiogenesis) (Mirzaei et al., 2018). Intriguingly, miR expression in brain is mirrored by differential expression of miRs implicated in endothelial cell and vascular function, erythropoiesis, angiogenesis, neural function, and hypoxia, even several months after the onset of stroke. This suggests that miRs play role in the response to cerebral ischemia and can serve as clinical biomarkers of stroke and post-stroke injury/recovery. In stroke and post-stroke remodeling of the BBB and NVU, a spectrum of miRs are involved varying with time, cell-specific target and recovery process. Importantly, due to the ability of miRs to target multiple molecules, often one miR is found to be on the interface of ongoing angiogenesis, inflammation, and/or oxidative stress.

Several miRs play a critical role in regulating post-stroke inflammation at the NVU. miR126 is highly expressed and considered as endothelial specific. It is one of the most heavily studied miRs and it is a potent regulator of vascular inflammation. miR126 modulates/downregulates expression of an adhesion molecule, VCAM, and controls leukocyte extravasation into brain (Yuan et al., 2016). Similarly, miR31 and miR17-3p regulate/antagonize the expression of E-selectins and ICAM-1 reducing neutrophil adhesion and post-stroke leukocyte infiltration (Staszel et al., 2011). miR98 and let-7g decrease leukocyte adhesion to and migration across the BBB (Rom et al., 2015). Two other miRs, miR155 and let-7, also have anti-inflammatory effects. They regulate expression of molecules important in stroke recovery including IL4, IL10, and BDNF in microglia and down-regulate iNOS and IL6 (Rom et al., 2015; Xiang et al., 2017; Greco et al., 2018; Lu et al., 2018). Intriguingly, miR155 has proinflammatory effects in the brain after stroke. It regulates macrophage signaling, differentiation and proinflammatory phenotype by diminishing the balance between suppressor of cytokine signaling 1 (SOCS-1) protein, and upregulating of inflammatory molecules such as iNOS (Lopez-Ramirez et al., 2014; Liu Y. et al., 2015; Cerutti et al., 2016). miR155 also stimulates expression of TNFα and IL1β via NFκB and toll-like receptor 4 (TLR4), and downregulation of myeloid differentiation primary response gene (MyD88) (Tarassishin et al., 2011). Furthermore, administration of miR155 antagomir decreased post-stroke inflammation, reduced neuronal apoptosis and improved neurological deficits (Pena-Philippides et al., 2016). Similar to the effect of miR155, an antagomir for miR210 decreased expression of proinflammatory cytokines IL6, TNFα, IL1β, CCL2, and CCL3 reducing the neurological impairment, putting miR210 as potent regulator of inflammation during stroke recovery (Huang et al., 2018).

Intriguingly, miRs are also associated with inflammasome activity. The inflammasome is a cytosolic complex that plays a role in ischemic stroke by promoting inflammatory and cell death mechanisms. miR223 has a role in suppressing the NLRP3 inflammasome by binding to its 3′ UTR sites and consequent inhibition of IL1β and caspase-1 activity, reducing brain edema and improving neurological score (Maitrias et al., 2017). The miRs, miR124, miR132a and 149-5a also have anti-inflammatory effects after stroke by inhibiting CCAAT/enhanced-binding protein (C/EBP-alpha) and downstream factor PU.1, promoting microglia quiescence, and suppressing activation of microglia and consequently reducing the expression of proinflammatory cytokines and suppressing proinflammatory mediators like NFκB, TNFα, and IL6 (Wanet et al., 2012; Yu et al., 2017; Wan et al., 2018).

Nuclear factor erythroid 2-related factor 2 (Nrf2) is a master regulator of anti-oxidant defense mechanisms that plays a critical role in limiting ischemia-induced brain injury, including BBB disruption (Alfieri et al., 2013). Papp et al. (2012) examined the Nrf2 interactome and identified 85 miRs involved in post-transcriptional regulation of Nrf2 (Wang et al., 2016). After transient MCAO in mice, Wang et al. (2016) found a marked increase in miR93 and that a miR93 antagomir reduced brain injury. The antagomir markedly increased brain Nrf2 levels as well as hemeoxygenase-1, a protein regulated by Nrf2; i.e., miR93 acts as a repressor of Nrf2, and antagonizing its actions may be a therapeutic approach for stroke (Wang et al., 2016).

Another miR, miR1 is indicated to have an important role in regulating neurotropic factors, apoptosis, neurogenesis and the generation of synapses (Talebi et al., 2019). Indicated as being an important target in treating neurodegenerative diseases as well myocardial infraction, miR1 suppression has a protective effect in stroke by reducing BBB permeability and brain edema and improving neurological deficits (Talebi et al., 2019). Similarly, miR15a is considered as a master regulator of cerebral vascular dysfunction after ischemic stroke. Intriguingly, after ischemic episodes, PPARγ and PPARd modulate miR15a expression at the cerebrovasculature, reducing injury and enhancing vascular recovery (Yin et al., 2010, 2013). Therefore, targeting miR15a is a promising strategy for protecting the BBB and the NVU after stroke.

Other processes regulated by miRs in post-stroke recovery include angiogenesis and BBB stability. In post-stroke associated angiogenesis, the miR expression profile is associated with the degree of post-stroke angiogenisis. Some miRs regulating angiogenic processes include miR225, miR335, miR139-5p, miR203, miR708, miR193 and miR494, which are predominantly downregulated, as well as miR224, miR210, miR204, miR322, miR100, miR450a, miR322, miR331 and miR101a which are upregulated in post-stroke angiogenesis (Lou et al., 2012; Yin et al., 2015). These miRs regulate expression of fibroblast growth factor (bFGF) and vascular endothelial growth factor (VEGF) in endothelial cells (miR15a and miR126), tube formation (miR139, miR335, and miR107), cell migration (miR210), targeting Dicer-1 and consequently VEGF (miR107) (Tome et al., 2011; Ye et al., 2014; Li et al., 2015; Zhu et al., 2017). One recent study indicated that functional polymorphism in the 3′ UTR site of angiopoietin-1 gene affects the binding sites of miR211, and can reduce the risk of stroke occurrence, while miR129-5p ameliorates inflammation and promotes revascularization after stroke (Chen et al., 2010; Li X.Q. et al., 2017).

Evidence indicates that several miRs are critical in regulating BBB integrity after stroke. A miR491-5p decrease leads to increased risk of cerebral ischemia and worsening stroke-induced BBB disruption by directly regulating expression of MMP9 (Yan et al., 2011). Suppression of miR155 2 days after ischemia in an experimental mouse stroke model decreased vascular leakage and promoted revascularization through stabilization of TJs and preservation of the BBB (Greco et al., 2018). Two recent studies showed that inhibiting miR130a in cerebral ischemia reduced BBB permeability, brain edema and enhanced neurological function by targeting Homeobox1 (Chen et al., 2010; Saito et al., 2011). A recent study by Yao et al. (2018) showed that miR21 suppresses MAP2K3 expression in brain endothelial cells regulating barrier permeability and exacerbation of brain edema and ischemic injury. miR149-5p also regulates BBB stability and NVU remodeling after cerebral ischemia. In addition, a miR149 polymorphism was reported to be associated with ischemic stroke pathogenesis (Jeon et al., 2013). A recent study by Wan et al. (2018) reported that miR149-5p targets the sphingosine phosphate receptor 2 (S1PR2) and regulates pericyte migration and N-cadherin expression. Thus, treatment with an agomir of miR149-5p attenuated BBB permeability and improved outcomes after transient MCAO.

**FIGURE 2:**
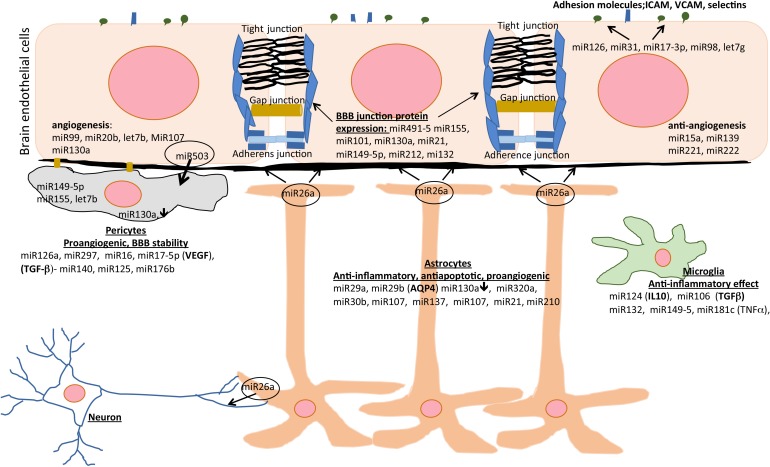
The effects of miRs on different cell types of the NVU during post-stroke recovery. Different miRs modulate inflammation, angiogenesis, apoptosis, and blood-brain barrier (BBB) integrity and represent therapeutic area to enhance post-stroke recovery. AQP4, aquaporin-4; ICAM, intercellular adhesion molecule; IL-10, interleukin-10; TGF-β, transforming growth factor beta; VCAM, vascular cell adhesion molecule; VEGF, vascular endothelial growth factor.

A number of miRs are considered as cell specific. In the line with that, miRs mostly involved in regulating endothelial cell function include miR126, miR17-92, miR23-27-24, miR222-22, miR99, miR20b, miR101, and Let-7b (Mishra and Singh, 2013). Astrocytes at the NVU are regulated by miR29b, that regulates AQP4 expression, as well as by miR130a, and miR320a (Sepramaniam et al., 2012; Song et al., 2015; Wang et al., 2015). miR130a is expressed in brain endothelial cells and pericytes. It regulates occludin expression via HoxA5 as well MMP2 and -9 (Wang et al., 2018). Intriguingly, miR130a is downregulated after ischemic conditioning in astrocytes and pericytes which may indirectly affect TJ complex structure and brain endothelial barrier permeability. A summary of miR activities at the BBB and post-stroke NVU recovery is presented in Figure 2.

An important aspect of miRs in stroke and post-stroke recovery is whether changes in their circulating levels reflect the degree/type of injury and potential recovery. For example, blood levels of miR30a and miR126 in patients with ischemic stroke are reduced for 24 weeks (Long et al., 2013). Another example is a differential pattern of expression across different stroke subtypes for a miR, let-7b. Let-7b was lower in stroke patients of a large atherosclerosis subtype compared to non-stroke patients, whereas in other ischemic stroke subtypes, let-7b was higher than in healthy individuals (Long et al., 2013). Thus, screening of different types of miRs may serve as a useful tool to assess the risks and prognosis of specific stroke subtypes and stroke recovery.

It should be noted that while miRs have actions in the cells where they are produced, they can also have effects on other distant cells. One mechanism is via the cellular release and uptake of exosomes. That type of extracellular vesicle contains a wide range of molecules, including miRs. Exosomes are being examined both as biomarkers for stroke and as a therapeutic modality (Hong et al., 2018). Exosomes are produced and taken up by all cells of the NVU, having an important role in cellular crosstalk, including in stroke (Zagrean et al., 2018), potentially via the effects of miRs. Thus, Xu et al. recently found that exosomes released by neurons regulate BBB permeability in zebrafish (Xu B. et al., 2017; Zagrean et al., 2018). Those exosomes contained miR132 and delivery of that miR regulated adherens junction function in the cerebral endothelium (Xu B. et al., 2017).

Apart from the role of endogenous exosomes, there is interest in using exogenous exosomes as a delivery platform, including loading such exosomes with select miRs (Beuzelin and Kaeffer, 2018). Thus, bacterial-based mini-cells have been loaded with a miR16-based mimic and given to patients with malignant pleural mesothelioma (van Zandwijk et al., 2017). Such approaches might be used to limit stroke-induced NBVU/BBB injury or enhance recovery.

The above description suggests that miR agonists (agomirs) and antagonists (antagomirs) may be attractive targets for reducing ischemia-induced BBB and NVU dysfunction and improving post-stroke recovery, particularly as each miR many affect multiple processes. There are, however, hurdles that will need to be overcome. The ability of miRs to affect multiple pathways suggests that special attention may be required for potential side-effects of treatment. There is also the question of delivery of the therapeutic to the appropriate target. Targeting the cerebral endothelium may be easier than targeting other cells of the NVU where the miR, agomir or antagomir would have to cross the BBB. Progress has been made in adeno-associated viruses that will cross the BBB that might serve as a route for delivery.

## Other Non-Coding RNAs

Currently, most studies have focused on the role of small ncRNA in regulating the BBB and NVU function in stroke. However, several recent studies have identified stroke-responsive piRNAs and lncRNAs (Alishahi et al., 2019). LncRNAs are not junk RNA but act as functional regulator elements in epigenetic regulation. They regulate gene function at different levels including but not limited to chromatin modification, and transcriptional and post-transcriptional mechanisms.

LncRNAs are longer than 200 nucleotides and have less sequence conservation than short ncRNA. Similar to short ncRNAs (miRs), lncRNAs regulate many important physiological processes including apoptosis, autophagy, immune response, and angiogenesis. LncRNAs may act as competitors to endogenous RNAs, acting as miR sponges or antagomirs (Alishahi et al., 2019). Recently, lncRNAs have been identified as being involved in human diseases. One lncRNA, Malat1 is involved in protecting against cerebrovascular ischemic injury. It is upregulated in brain endothelial cells by ischemia-like conditions and reduces injury. Malat1 acts by being a sponge for miR26b, regulating brain endothelial cell autophagy and survival under ischemic conditions (Li Z. et al., 2017).

## Conclusion

The epigenetic mechanisms and the non-coding RNAs involved in BBB and NVU changes in stroke and stroke recovery are slowly emerging. That information holds great promise for better understanding of stroke pathology but also for developing new strategies for successful stroke recovery. In the future, both epigenetic factors and non-coding RNAs may have dual roles, as therapeutic targets and as biomarkers mirroring NVU injury and recovery processes. Epigenetic mechanisms are attractive targets as they affect multiple pathways. Histone deacetylase inhibitors are already used clinically for some disease states and more HDAC subtype-specific inhibitors are being developed. While the importance of miRs in a wide variety of disease states is recognized, there are still hurdles to cross in translating that to the clinic, particularly for neurological conditions such as stroke. However, the pleiotropic effects of epigenetic mechanisms and non-coding RNAs make them attractive and exciting therapeutic targets for limiting post-stroke NVU injury and enhancing recovery.

## Author Contributions

All authors contributed to the writing, illustration, and reviewing of the manuscript.

## Conflict of Interest Statement

The authors declare that the research was conducted in the absence of any commercial or financial relationships that could be construed as a potential conflict of interest.
